# Function inference of million-scale microbiomes using multi-GPU acceleration

**DOI:** 10.1128/spectrum.02072-25

**Published:** 2025-11-07

**Authors:** Yu Zhang, Minan Wang, Yangyang Sun, Hao Gao, Xiaoquan Su

**Affiliations:** 1 College of Computer Science and Technology, Qingdao University 12593, Qingdao, China; The Chinese University of Hong Kong, Hong Kong, Hong Kong

**Keywords:** microbiome, function inference, GPU, parallel computing

## Abstract

Amplicon sequencing enables taxonomic profiling of microbial communities but offers limited insight into their functional potential. Existing tools such as Phylogenetic Investigation of Communities by Reconstruction of Unobserved States (PICRUSt2) infer functions through phylogenetic placement and ancestral state reconstruction; however, these methods are computationally intensive and inefficient for large-scale data sets. To address these challenges, we introduce microbiome graphics processing unit (GPU)-based function inference (MGFunc), an ultra-high-throughput framework for microbiome functional inference leveraging multi-GPU acceleration. MGFunc transforms functional prediction for amplicons into standardized matrix multiplication using a pre-constructed genomic content network. It further integrates split data loading, matrix partition, and dynamic scheduling across multiple GPUs, enabling scalable, batch-wise analysis of millions of samples under limited GPU memory and system random access memory (RAM). Compared to PICRUSt2, MGFunc achieves speedups of up to several hundred thousand times, completing the functional interpretation of one million samples within one minute by four GPUs on a single server. This work provides a highly efficient and low-latency solution for ultra-large microbiome data sets functional inference, paving the way for global-scale microbiome studies. The MGFunc software is freely accessible at https://github.com/qdu-bioinfo/MGFunc.

**IMPORTANCE:**

Understanding what microbes do—their functions—is essential for studying health, disease, agriculture, and the environment. While cost-effective sequencing methods like 16S rRNA gene analysis are widely used, they do not directly reveal microbial functions. Existing tools that predict these functions from 16S data are often too slow for today’s large studies involving hundreds of thousands of samples. In this work, we developed microbiome graphics processing unit (GPU)-based function inference (MGFunc), a new method that predicts microbial functions quickly and accurately by using GPUs and a streamlined mathematical approach. MGFunc can analyze over one million samples in under a minute, making it one of the fastest tools available. This enables researchers to study the functional potential of microbial communities on a truly global and population scale.

## INTRODUCTION

Microbial communities play a fundamental role in maintaining ecosystem stability, promoting host health, and supporting agricultural productivity (1, 2). Understanding their functional roles, such as nutrient cycling, toxin degradation, and metabolic interactions, is essential for advancing both environmental and biomedical research (3, 4). A primary objective of microbiome studies is the functional annotation of microbial communities, which aims to characterize their metabolic capacities, ecological relationships, and adaptive responses to environmental changes (5). As sample sizes continue to expand, the ability to capture the complex interactions between microbiomes and their hosts improves. Large-scale collaborative initiatives, such as the Earth Microbiome Project (6) and the American Gut Project (7), have systematically explored the associations between the microbiome and various influencing factors, highlighting the tremendous potential of large-scale microbiome data sets for uncovering ecological and biomedical insights (8).

Shotgun metagenomic sequencing has become the “gold standard” for functional profiling, which can be achieved by mapping sequence data onto curated functional databases (9). Despite its high resolution and comprehensive coverage, shotgun metagenomics remains costly and requires high-quality DNA, hindering its widespread use, particularly in large-scale or resource-constrained studies (10). In contrast, tools such as PICRUSt2 have been developed to predict functional profiles from affordable amplicon sequencing (e.g. 16S rRNA gene) using phylogenetic inference and ancestral state reconstruction (11, 12). Nevertheless, such approaches are computationally intensive and do not scale efficiently with growing data set sizes.

In this work, we present microbiome graphics processing unit (GPU)-based function inference (MGFunc), a novel ultra-high-throughput method for microbiome functional inference based on amplicon data. By leveraging a pre-constructed genomic content network (GCN) and multi-GPU acceleration, MGFunc reformulates functional prediction as standardized matrix multiplication, achieving significant improvements in scalability and performance (13). This work provides a robust and scalable alternative to traditional methods, enabling accurate functional inference across millions of microbiome samples.

### Method

#### Function inference via GCN and matrix multiplication

**Fig 1:**
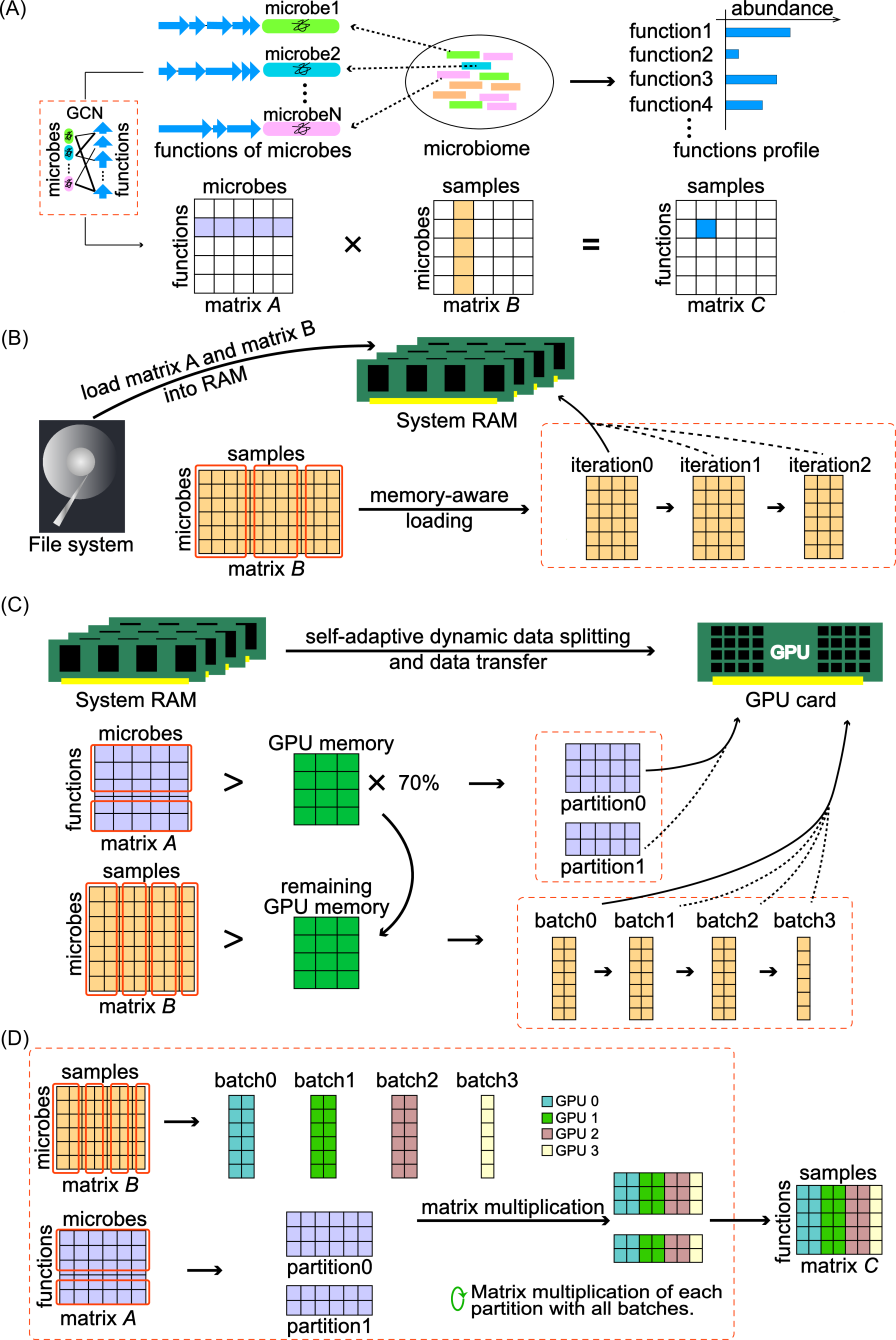
The overall scheme of MGFunc. (**A**) Functional inference workflow: the microbiome’s functional composition is estimated via matrix multiplication of the function–microbe matrix *A* and the microbe–microbiome matrix *B*. (**B**) Memory-aware loading strategy for large data sets: when the matrix *B* exceeds available system RAM, it is divided into iterations and processed iteratively. (**C**) GPU memory management and matrix splitting. MGFunc pre-allocates 70% of GPU memory for matrix *A. A* is partitioned if it exceeds such threshold; otherwise, it is fully loaded and releases the remaining pre-allocated memory. All the available GPU memory is then used to batch matrix *B* for processing. (**D**) Multi-GPU parallel execution: in multi-GPU mode, the same partition of matrix *A* is loaded into all GPUs, while matrix *B* is divided into batches by samples and distributed across GPUs for concurrent matrix multiplication.

MGFunc begins by pre-constructing a bipartite GCN that links full-length 16S rRNA genes of reference microbes to their annotated functional profiles derived from whole-genome sequences (refer to the *GCN construction and reference databases* section for details). This network yields a function–microbe matrix *A*, where each element *A*[*i*][*k*] denotes the normalized copy number of functional gene *i* in microbe *k* (Fig. 1A).

To apply this to amplicon sequencing data, we map each operational taxonomic unit or amplicon sequence variant (ASV) to reference full-length 16S rRNA genes (14) (refer to the *Microbiome profiling* section for details). This generates a microbe–microbiome matrix *B*, where each element *B*[*k*][*j*] represents the sequence count of microbe *k* in sample *j*. Once an amplicon sequence is matched to a reference microbe, its functional profile can be inferred through the corresponding entries in matrix *A*.

The predicted richness of function *i* in microbiome sample *j*, represented as C[*i*][*j*], is computed by standard matrix multiplication, where *n* denotes the total number of microbes across all samples (equation 1):


(1) $$C\left[i\right]\left[j\right]=\sum_{k=1}^{n}A\left[i\right]\left[k\right]*B\left[k\right]\left[j\right]$$

This operation yields the function-sample matrix *C*, which estimates the overall functional composition of each sample (Fig. 1B).

### Matrix multiplication accelerated by GPU–CPU hybrid parallelization

Matrix multiplication is a highly parallelizable operation, ideal for GPU acceleration due to its reliance on large numbers of independent multiply-and-accumulate tasks. Modern GPUs, equipped with thousands of processing cores, can perform these tasks concurrently, making them well suited for large-scale microbial functional prediction involving high-dimensional matrices.

To harness this parallelism, MGFunc implements its computing kernel using NVIDIA CUDA (compute unified device architecture) (15) or heterogeneous-computing interface for portability (HIP) (16), which provides optimized linear algebra operations for GPU architectures. These technologies accelerate matrix multiplication while simplifying low-level GPU programming. In addition, MGFunc employs OpenMP multithreaded parallelism (17) on the CPU side to manage preprocessing tasks such as sample partitioning, matrix blocking, and intput/output (I/O) operations (refer to the *Memory-aware loading strategy for RAM management* section for details). This hybrid CPU–GPU design minimizes data preparation time and maximizes overall pipeline efficiency.

### Memory-aware loading strategy for RAM management

Initially, both function–microbe matrix *A* and microbe–microbiome matrix *B* are loaded from the file system to the system RAM for processing via I/O operations. The size of the matrix *A* is generally manageable, as it is constrained by the number of known microbial taxa and functional gene categories (refer to the *GCN construction and reference databases* section for details). For example, a function–microbe matrix *A* constructed from the Greengenes (18) database for KEGG ortholog (KO) (19) occupies approximately 5.2 GB, making it feasible to load entirely into system RAM. In contrast, the matrix *B* can become extremely large when processing data sets with millions of samples, often exceeding available system RAM.

Thus, MGFunc implements a memory-aware loading strategy that enables efficient, iterative processing of large-scale data sets without exhausting RAM. Users can specify the maximum number of samples to load per iteration. MGFunc then loads each iteration of samples from matrix *B*, records the current loading position, performs the matrix multiplication as described previously, and proceeds iteratively until all samples have been processed (Fig. 1B; also refer to *Recommended iteration size for memory-aware loading* section for details). This strategy effectively balances memory usage and computational throughput, allowing MGFunc to process ultra-large-scale microbiome data sets on systems with limited RAM capacity.

### Self-adaptive dynamic data splitting for GPU ultra-scale processing

Then loaded function–microbe matrix *A* and microbe–microbiome matrix *B* are transferred from RAM to GPU onboard memory via the system bus. In large-scale functional prediction tasks, the matrices may also exceed the available memory of a single GPU. To overcome this limitation, we designed a self-adaptive dynamic data splitting method for GPU computing (Fig. 1C).

At runtime, MGFunc automatically detects the total available GPU memory and reserves a small fraction for intermediate variables and buffers. The remaining GPU memory is dynamically allocated for matrix loading, with a maximum of 70% pre-allocated for matrix *A*. If matrix *A* exceeds this threshold, it is partitioned along the microbe dimension into manageable segments; otherwise, it is transferred in its entirety to GPU memory and releases the pre-allocated space. Then, matrix *B* is dynamically divided along the sample dimension based on the actual available GPU memory size and loaded in batches for multiplication with the current partition of matrix *A*. This strategy ensures that matrix *A* is loaded entirely or with minimal partitioning to reduce redundant data transfers, while dynamically optimizing the memory usage for matrix *B* to enable efficient large-scale inference under memory constraints.

### Multi-GPU scheduling and task distribution

MGFunc supports both single-GPU and multi-GPU execution modes (Fig. 1D). In single-GPU mode, matrix multiplication is performed as described in Section 2.4. In multi-GPU mode, the user specifies the number of available GPUs. The same partition of the function-microbe matrix *A* is simultaneously loaded onto all GPUs. The microbe–microbiome matrix *B* is then divided along the sample axis and distributed across GPUs, allowing concurrent computation of different sample partitions. Once all GPUs complete their respective batch multiplications, the next batch of matrix *A* is loaded, and the process is repeated until the full matrix product is computed. This scheduling strategy enables MGFunc to efficiently scale functional inference tasks across multiple GPUs, significantly accelerating large-cohort microbiome analyses.

## RESULTS

### Datasets and testing environment

To comprehensively evaluate the performance of MGFunc, we compared its computation time against PICRUSt2 (only supports single-thread mode for determining gene family abundance) and Parallel-Meta Suite (PMS) (20) function prediction (supports CPU-based multi-thread parallel computing) across varying sample sizes. For fair benchmarking, simulated microbiome samples were used, each containing the same number of features: 99,315 microbial taxa and 13,839 KO functional categories. All tests were performed on a single rack server, and detailed specifications of the testing environment are provided in Table 1.

**TABLE 1:** Experiment environment and configuration

Feature		Configuration
Hardware	CPU	2 × Hygon C86 7375 (64 cores, supports 128 threads)
	GPU	4 × NIVIDA RTX 4090 GPUs, each with 16,384 cores and 24 GB onboard memory
	RAM	256 GB DDR4 ECC
Software	Operating system	NFSChina Server 4.0
	MGFunc	Version 1.0, compiled by CUDA 12.2
	PMS	Version 3.7.2, compiled by g ++ 8.3.1
	PICRUSt2	Version 2.4.2

### Computational efficiency and speedup

**Fig 2:**
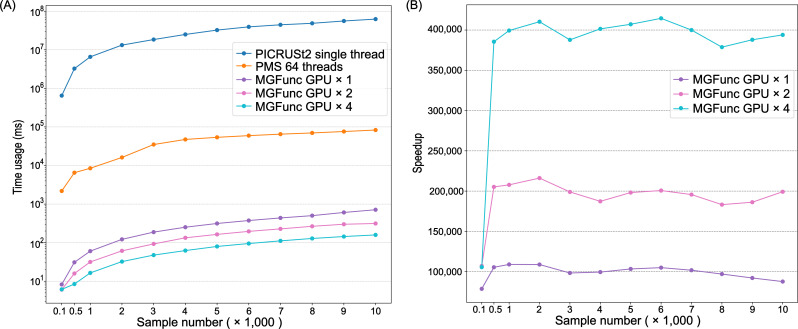
Computation time and speedup ratio of MGFunc for sample sizes below 10,000. (**A**) Computation time (in milliseconds) of MGFunc, PMS, and PICRUSt2 across varying sample sizes. MGFunc demonstrates consistently low runtimes regardless of GPU count. (**B**) Speedup ratios of MGFunc using 1, 2, and 4 GPUs compared to PICRUSt2, calculated as the ratio of PICRUSt2’s computation time to that of MGFunc. Results show substantial performance gains and near-linear scaling with additional GPUs. Source data are available in Table S1.

We benchmarked the computational time of MGFunc, PMS, and PICRUSt2 for determining gene family abundance across a range of sample sizes (Fig. 2A). MGFunc consistently delivered rapid performance, completing predictions in under one second even with 10,000 samples, regardless of whether 1, 2, or 4 GPUs were used. In comparison, PMS and PICRUSt2 required over 80 s and approximately 60,000 seconds (~18 h), respectively, to process the same data set. These results underscore the dramatic acceleration achieved by MGFunc’s GPU-accelerated architecture.

To quantify performance gains, we calculated the speedup ratio as the computation time of PICRUSt2 divided by that of MGFunc. As shown in Fig. 2B, MGFunc achieved approximately 100,000× speedup with a single GPU, 200,000× with two GPUs, and up to 400,000× using four GPUs. These results confirm MGFunc’s highly scalable performance and efficient parallelization. The near-linear scaling with GPU count demonstrates effective workload distribution with minimal overhead.

Overall, MGFunc significantly reduces runtime for microbiome functional inference and efficiently scales with increasing GPU resources, offering a robust solution for high-throughput analyses.

### Performance on ultra-large-scale data

**Fig 3:**
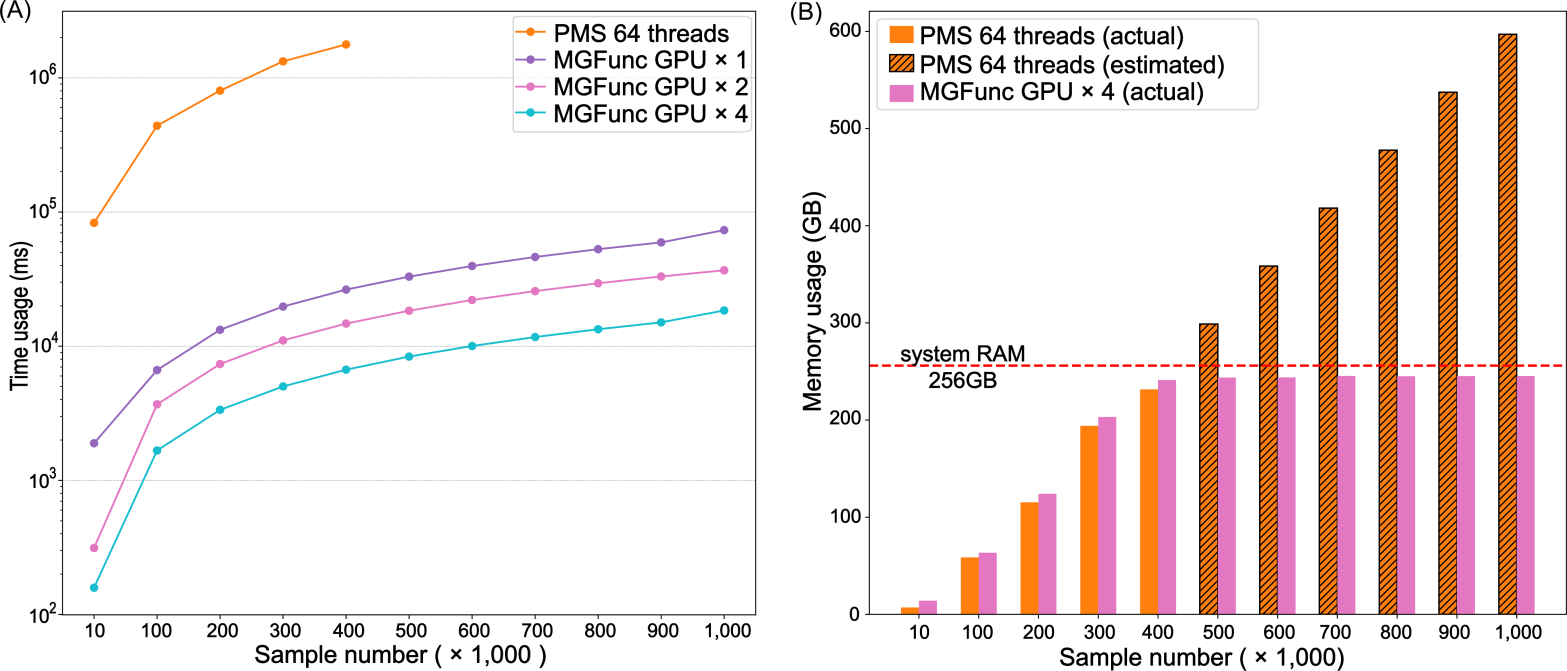
Comparison of computation time and system RAM usage as sample size increases to hundreds of thousands. (**A**) Comparison of the time required to complete large-scale sample computations between the PMS and MGFunc. (**B**) Comparison of system RAM usage during large-scale sample computations between the PMS and MGFunc. The hatched segments indicate estimated memory usage for PMS by linear regression (R^2 ^= 0.9915) when exceeding the 256 GB server memory limit. Source data are available in Tables S2 and S3.

To evaluate MGFunc’s performance on ultra-large data sets, we progressively increased the sample size up to one million. With 256  GB system RAM and iteration sample size *n *= 400,000 (refer to the *Recommended iteration size for memory-aware loading* section for details), MGFunc successfully processed these larger data sets and completed the computation for one million samples within one minute using four GPUs (Fig. 3A). In contrast, when the sample number exceeded the MGFunc iteration size, the PMS was unable to complete the computation due to system RAM limitations (Fig. 3B). Based on extrapolated runtimes, PICRUSt2 would take approximately 70 days to process one million samples under the same conditions.

In addition, we further estimated the memory demands of the PMS at large sample sizes by a linear regression based on its measured computation times at smaller scales. The results (Fig. 3B) suggested that PMS would require approximately 300 GB of memory to process 500,000 samples and approximately 600 GB to process one million samples, exceeding the available system RAM and thus explaining its computational failure at these scales.

These results demonstrate that MGFunc can flexibly manage system RAM and efficiently perform large-scale functional prediction through its iterative loading and computation mechanism.

### Consistency to PICRUSt2

To evaluate the consistency between MGFunc and PICRUSt2, we analyzed 569 real microbiome samples spanning seven distinct ecosystem types, including human-associated, animal-associated, and various natural environments (Table 2; Table S4). All data sets were obtained from publicly available studies. For each sample, microbial functional profiles were predicted based on KO using both MGFunc and PICRUSt2.

**TABLE 2:** Sources of the selected environments. Detailed information of microbiome samples is available in Table S4

NCBI project ID	Type	Sample number
ERP012611	Feces	175
SRP163178	Nose	71
ERP113175	Lung	70
SRP026653	Oral	92
SRP079007	Fish	89
SRP090623	Lake	40
SRP182082	Cropland	32

**Fig 4:**
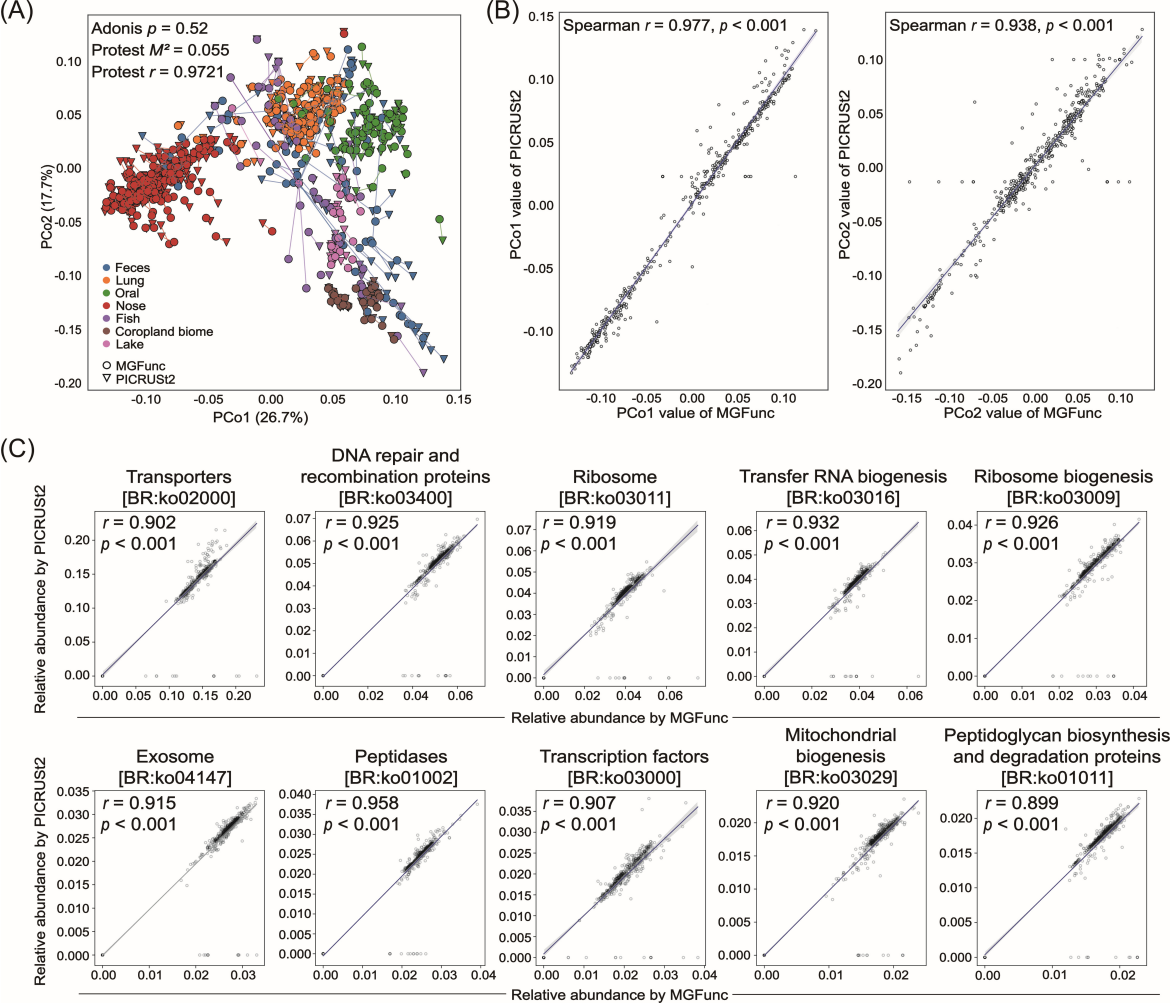
Consistency between MGFunc and PICRUSt2. (**A**) PCoA plots for seven different environments. Circles represent MGFunc, and inverted triangles represent PICRUSt2, and colors indicate different environments. Profiles derived from the same sample were linked by lines. (**B**) Each dot represents a sample, where the x-axis denotes the PCo values obtained from MGFunc and the y-axis denotes the PCo values obtained from PICRUSt2. (**C**) The abundances of top ten abundant KEGG BRITE Level three pathways were compared between MGFunc and PICRUSt2, with MGFunc values plotted on the x-axis and PICRUSt2 values on the y-axis. Source data are available in Tables S4 to S6.

Principal coordinate analysis (PCoA) (21) based on the hierarchical Meta-Storms (HMS) distance (22) revealed that the functional compositions inferred by MGFunc and PICRUSt2 exhibited highly similar distribution patterns across the different environments (Fig. 4A). Both PCo1 and PCo2 values showed strong and statistically significant correlations across all samples (Fig. 4B), supporting high concordance in overall functional structure. Additionally, a PERMANOVA (Adonis) test confirmed no significant difference between the functional community structures predicted by the two methods (*P *= 0.52), further demonstrating methodological agreement (23). Furthermore, we performed a Procrustes analysis (24, 25) to quantitatively assess the overall concordance of the predicted functional profiles between MGFunc and PICRUSt2. The results revealed a remarkably high degree of concordance, with *M² *= 0.055 and *r *= 0.9721.

Concordance was further examined at a more granular level by comparing KO annotations within KEGG BRITE Level 3 pathways (26). Across the top 10 most abundant functional categories—such as transporters, ribosome biogenesis, DNA repair, and transcription factors—MGFunc and PICRUSt2 produced highly consistent predictions. Scatter plots of predicted KO abundances showed strong linear relationships without systematic bias, reinforcing the reliability of MGFunc’s predictions (Fig. 4C).

**Fig 5:**
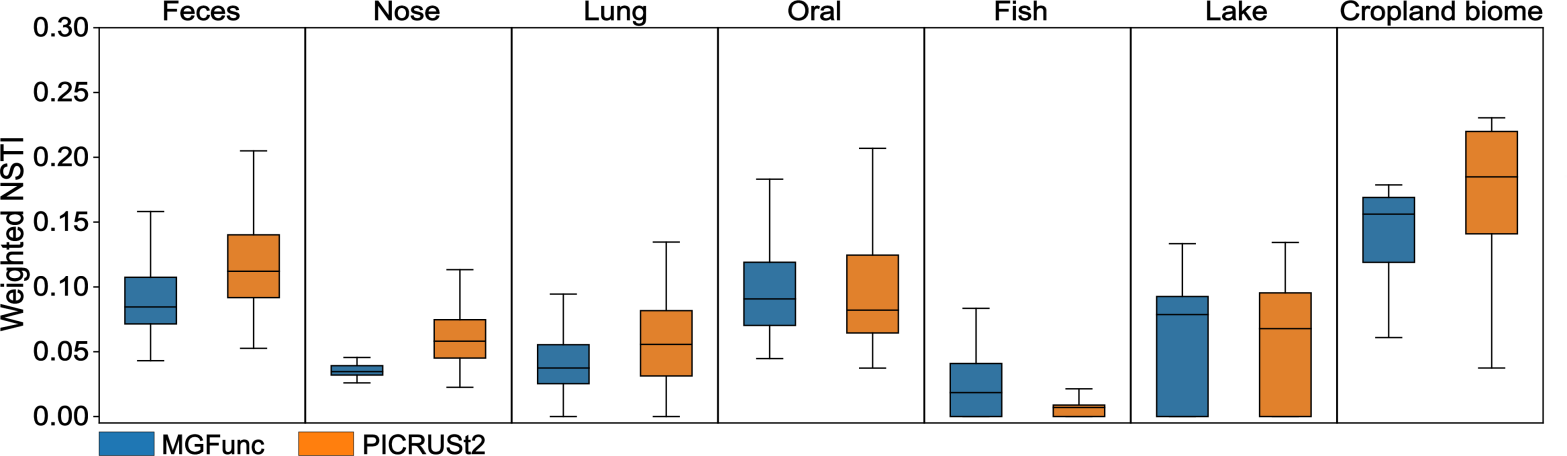
Weighted NSTIs. Boxplots of weighted NSTI values predicted by MGFunc and PICRUSt2 across different environments. Source data are available in Table S7.

Furthermore, we compared the weighted nearest sequenced taxon index (NSTI) values of all samples as estimated by both methods. NSTI values reflect the phylogenetic proximity of observed microbes to sequenced reference genomes, thereby serving as a proxy for prediction confidence. Across all environments, MGFunc and PICRUSt2 exhibited similar NSTI value distributions, indicating comparable coverage and inference quality across diverse microbial communities (Fig. 5).

Furthermore, we compared the NSTI values of all samples as estimated by both methods. NSTI values reflect the phylogenetic proximity of observed microbes to sequenced reference genomes, thereby serving as a proxy for prediction confidence. Across all environments, MGFunc and PICRUSt2 exhibited similar NSTI value distributions, indicating comparable coverage and inference quality across diverse microbial communities (Fig. 5).

## DISCUSSION

The increasing scale and complexity of microbiome studies have created a pressing need for tools that can deliver both high-throughput and accurate functional inference. While shotgun metagenomics remains the gold standard for direct functional profiling, its high cost and computational burden limit its feasibility in large-scale projects. Amplicon-based functional prediction tools such as PICRUSt2 provide a cost-effective alternative but struggle with scalability and computational efficiency, especially as data set sizes grow into the millions.

In this study, we introduced MGFunc, a GPU-accelerated, matrix-based framework designed to address these limitations by reformulating functional prediction as standardized matrix multiplication. Leveraging a pre-constructed GCN, MGFunc decouples function inference from repeated phylogenetic placement or ancestral state reconstruction, dramatically reducing computational overhead. With the integration of multi-GPU parallelization, dynamic memory-aware batching, and efficient CPU–GPU scheduling, MGFunc achieves exceptional scalability—completing the functional profiling of one million microbiome samples in under one minute on a single server with four GPUs.

While MGFunc represents a major advancement in functional prediction throughput, it inherits the limitations of reference-based approaches, such as dependence on the completeness and representativeness of the underlying genome database. Continued updates to microbial genome repositories will be essential to maintain and improve prediction accuracy, particularly for understudied environments and rare taxa. In conclusion, MGFunc offers an ultra-fast, scalable, and accurate solution for microbiome functional inference based on amplicon data. By bridging the gap between computational efficiency and biological fidelity, MGFunc enables next-generation microbiome studies at an unprecedented scale and speed.

## MATERIALS AND METHODS

### GCN construction and reference databases

We constructed the GCN using the PICRUSt2 strategy: full-length 16S rRNA gene sequences were aligned to the PICRUSt2 reference database and placed in the phylogeny. Thus, the gene family copy numbers of each microbe were obtained through hidden state prediction (27). Specifically, this method employs phylogenetic inference based on a continuous-time Markov model, predicting gene content and nearest sequenced taxon index (NSTI) values with evolutionary distance-based weighting (closer genomes receive higher weight) for each microbe. In addition, genomes with identical 16S sequences were collapsed into a single cluster, and their gene family copy numbers were averaged to generate representative trait values. The resulting function–microbe matrix *A* provides the computational basis for MGFunc’s downstream predictions.

To ensure broad applicability and generalizability, we pre-processed five widely used 16S reference databases. The details of each database, including taxonomic scope, sequence count, and supported functional annotations, are summarized in Table 3.

**TABLE 3:** Reference databases of matrix *A*

Reference database	# of microbes	# of functions (KEGG ortholog)	Matrix *A* size
Greengenes (18)	99,322	13,839	5.12 GB
Greengenes2 (28)	331,269	13,839	17.08 GB
Silva (14)	152,265	13,839	7.85 GB
Refseq (29)	101,192	13,839	5.22 GB
RDP (30)	24,624	13,839	1.27 GB

### Microbiome profiling

For MGFunc-based functional profiling, raw 16S rRNA amplicon sequencing reads were denoised and clustered into ASVs, and then mapped to Greengenes database (v13-8) with a sequence similarity threshold of 0.99 by Parallel-Meta Suite (version 3.7.2) to generate the microbe–microbiome matrix *B*.

For PICRUSt2-based function profiling, raw 16S rRNA amplicon sequencing reads were processed using QIIME2’s reference-based clustering and Greengenes database (v13-8) to produce both taxa abundance tables and representative sequences (31). The representative sequences were subsequently input into PICRUSt2 to generate functional abundance profiles.

### Recommended iteration size for memory-aware loading

When the microbe–microbiome matrix *B* exceeds available system memory, it is recommended to enable MGFunc’s memory-aware loading strategy by specifying the maximum number of samples to load per iteration. To facilitate optimal use of system resources, we provide recommended sample sizes per iteration corresponding to different RAM capacities in Table 4.

**TABLE 4:** Recommended RAM usage for memory-aware loading

RAM size	Sample size per iteration
16 GB	10,000
32 GB	50,000
64 GB	100,000
128 GB	200,000
256 GB	400,000
384 GB	600,000
512 GB	800,000
640 GB	1,000,000

## Data Availability

The real microbiome data sets used in this study are publicly accessible via identifiers listed in Table 2 and Table S4. MGFunc was implemented in C++ with CUDA/HIP and OpenMP for GPU–CPU hybrid acceleration. The code is compatible with NVIDIA GPUs supporting CUDA 11.1 and higher, and AMD GPUs supporting HIP 5.7 and higher. It has been tested on CUDA 12.2 and HIP 24.04. Compilation was performed using g++ 8.3.1 with nvcc 12.2 or hipcc 24.04 under a Linux environment. The implementation supports both single-GPU and multi-GPU modes. The source code of MGFunc is freely available at https://github.com/qdu-bioinfo/MGFunc.

## SUPPLEMENTAL MATERIAL

Tables S1 to S7 spectrum.02072-25-s0001.xlsx Source data.

ASM does not own the copyrights to Supplemental Material that may be linked to, or accessed through, an article. The authors have granted ASM a non-exclusive, world-wide license to publish the Supplemental Material files. Please contact the corresponding author directly for reuse.
